# Supplementary material for: Variations in Genetic Diversity of Invasive Species Lithobates catesbeianus in China
Source: Animals (Basel). 2024 Apr 25;14(9):1287. doi: 10.3390/ani14091287 (PMC11083970; doi:10.3390/ani14091287)
Supplement: Supplementary file 1 [file animals-14-01287-s001.zip › animals-2961175-supplementary.pdf]

Supplementary Materials

Variations in Genetic Diversity of Invasive Species *Lithobates catesbeianus* in China

Jiaqi Zhang [1,2], Chunxia Xu [1], Supen Wang [1,†], Siqi Wang [1,2] and Yiming Li [1,2,3,*]

[1] Key Laboratory of Animal Ecology and Conservation Biology, Institute of Zoology, Chinese Academy of Sciences, 1 Beichen West Road, Chaoyang, Beijing 100101, China; zhangjiaqi2025@163.com (J.Z.); xuchunxia0413@sina.com (C.X.); frog2019@ahnu.edu.cn (S.W.); wangsiqi17@mails.ucas.ac.cn (S.W.)

[2] University of Chinese Academy of Sciences, Beijing 100049, China

[3] School of Life Sciences, Institute of Life Sciences and Green Development, Hebei University, Baoding 071002, China

[*] Correspondence: liym@ioz.ac.cn

[†] Current address: College of Life Science, Anhui Normal University, Wuhu 241008, China.

Figure S1. The optimal number of clusters (best K value) for microsatellites (A) and SNPs (B) was determined using the ΔK estimation method and implemented in STRUCTURE HARVESTER, as related to Figure 2 and Figure 4.

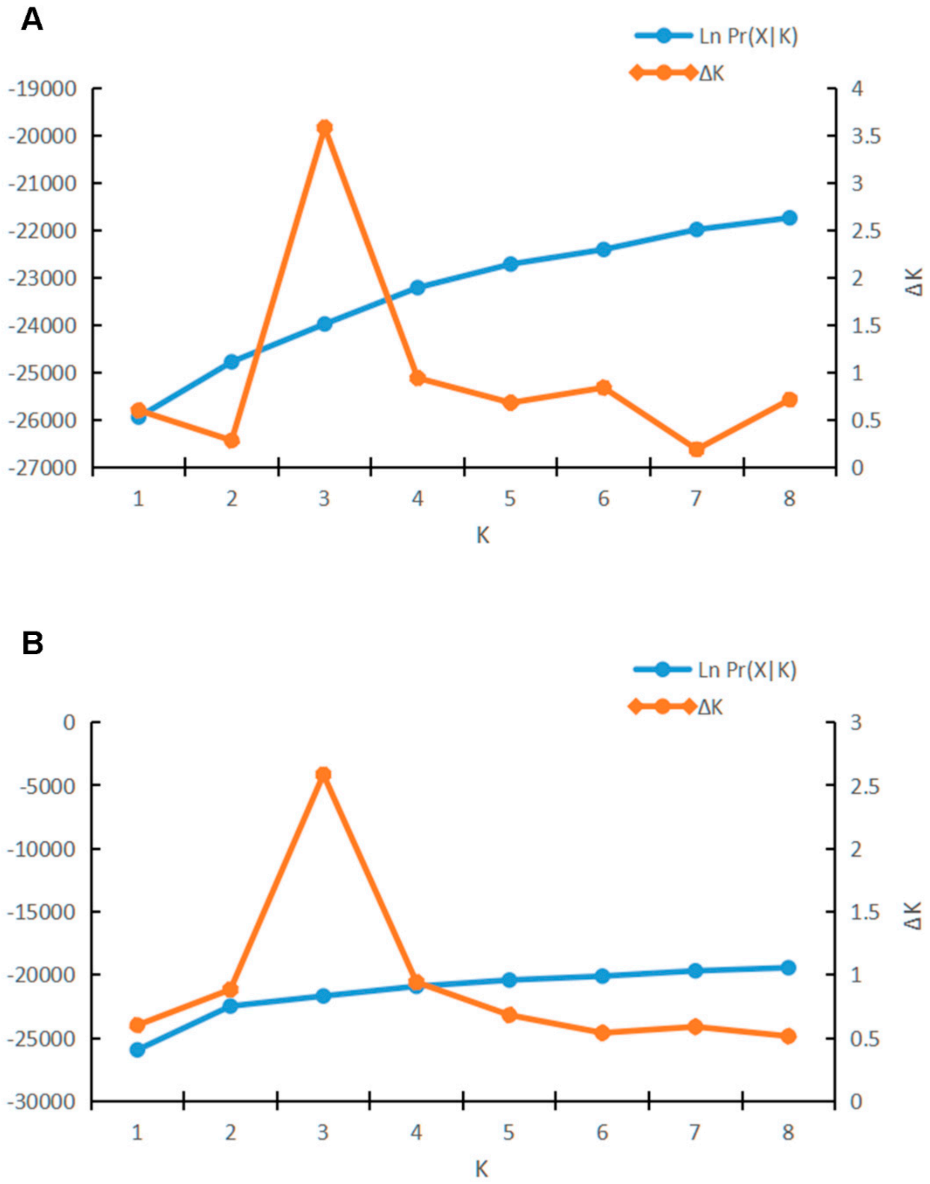

Table S1. Population sampling localities, GPS coordinates, sample sizes (n) and information on introduction history of 10 bullfrog populations in this study. Number of generations between first introduction and sample date was calculated based on one generation =3 year (the mid value of minimum year and maximum year at mature, Stringham et al 2021). Introduction pathway includes escapes from bullfrog or release by people.

site	Coordinates E	Coordinates N	Collection Date (year-month)	Introduction date (no. of generations)	sample sizes (n)	Introduction pathway
ZheJiang	122.256	30.006	2013-7	1994(8)	10	escape
YunNan	102.601	23.674	2014-8	1990(10)	10	escape
AnHui	118.508	31.151	2013-8	1985(12)	10	escape
Tibet	91.108	29.668	2017-9	1980(15)	10	release
TianJin	117.511	38.932	2014-8	1995(8)	10	escape
HuNan	112.93	28.138	2013-8	1959(22)	10	escape
ShanDong	116.296	35.957	2015-8	1988(11)	10	escape
SiChuan	117.283	34.69	2015-8	1985(12)	10	escape
Kansas	-98.505233	38.18017	2010-7	-	10	
California	-121.628064	38.559753	2010-7	-	10	

Table S2. Characteristics of the seventeen microsatellite loci used in the study.

Locus	Primer sequence (5'-3'')	Repeat motif	Length (bp)	*Ta* (°C)	Reference	Genbank #
Rcat3-2b	F: CACCCAACTAATTAGGATGGG<br>R: GGAATGGCATTCAGAGAGGG	(AC)2AT(AC)12	144	62	Austin *et al.* 2003	AY323934
RcatJ8	F: CCATAGGAATCAAAACAACCCTC<br>R: GGGATATGTGATGGACCCAAG	(GT)18	100	58	Austin *et al.* 2003	AY323931
RcatJ44b	F: AGGTTAATGAAGCTCGGCAG<br>R: GGAGGCATCATATCAGAGAG	(GT)22	93	60	Austin *et al.* 2003	AY323932
RcatJ54	F: TCATTACCACTGCCTTCTGC<br>R: TGCTGCTGTCCTATTGCTAG	(CA)18	183	60	Austin *et al.* 2003	AY323930
RcatJ21	F-CCCATCTTATCCTGTGTACT<br>R-CAAGCCTCCATCTCACCTTACC	(GT)25	160–166	60	Austin *et al.* 2003	AY323929
RcatJ11	F: CTCTTCCATTCACAACTGCAC<br>R: AGGCGGAGCATATATTGTGG	(CA)16C(CA)3	124	60	Austin *et al.* 2003	AY323928
BF194	F-CTGCTGTGGTATCTATGCTTTTT<br>R-CAACTGACATTTGCCCAACA	(TTTG)5	163–258	55	Austin *et al.* 2011	HQ439096
BF195	F-GGAGCCGAGTGTAACCTGAG<br>R-TGGACTCTATGCCAACACCA	(CA)33	163–215	55	Austin *et al.* 2011	HQ439097
BF249	F-CCATGGAATTTGCAGAATGA<br>R-GGCAAAAACCTGGCCTAAA	(CA)11	153–187	60	Austin *et al.* 2011	HQ439093
BF205	F-GGAACCCTCATTGAATTTCG<br>R-GCGTCCCGATACTTTTGACA	(TA)6(AC)8AGT(AG)36	105–187	53	Austin *et al.* 2011	HQ439094
BF299	F-TGTAGCAGCCAAATATCCTGAA<br>R-CTGTGTGTATGTGGCCAACC	(AGT)10	130–157	60	Austin *et al.* 2011	HQ439092
Htiger510	F: F9GTC - CCCATCCTGTTCTTGTGCAG<br>R: TACGGACTAAGCCTGGGATG	(AC)10	85–129	58	Sultana et al. 2014	AB911223
Htiger303	F: M13 - TTTCAGCCATGTCTGCTCAG<br>R: ACTGTTGGCGCATATTAAATCC	(TTA)12	169–274	58	Sultana et al. 2014	AB911228
Htiger2248	F: M13 - CAAGACAGACCGCAAAGACG<br>R: AGCACCATCAGTTTGTACAGTG	(AGAT)8	169–274	58	Sultana et al. 2014	AB911229
Htiger156	F: M13 - CCGGTGCACCTCTCTTATTC<br>R: GCAGGCGTTGGAAGTGAC	(AGC)12	135–192	58	Sultana et al. 2014	AB911231

Htiger1931	F: M13 - TGTTCTTGGTTTGGGTGCTG<br>R: ACCAGACACAACCACCCTAG	(TG)12	123–257	58	Sultana et al. 2014	AB911236
Htigr1409	F: F9AGG - ACACACCACCACTGCTCAG	(AC)4 ……(TG)10	101–124	58	Sultana et al. 2014	AB911222

1. Austin, J.D.; Davila, J.A.; Lougheed, S.C.; Boag, P.T. Genetic evidence for female-biased dispersal in the bullfrog, Rana catesbeiana (Ranidae). Mol Ecol 2003, 12, 3165-3172, doi:10.1046j.1365-294x.2003.01948.x.

2. Austin, J.D.; Gorman, T.A.; Bishop, D. Assessing fine-scale genetic structure and relatedness in the micro-endemic Florida bog frog. Conservation Genetics 2011, 12, 833-838.

3. Sultana, N.; Igawa, T.; Nozawa, M.; Islam, M.M.; Hasan, M.; Alam, M.S.; Khan, M.M.R.; Sumida, M. Development and characterization of 27 new microsatellite markers for the Indian bullfrog Hoplobatrachus tigerinus and its congeneric species. Genes & genetic systems 2014, 89, 137-141.

Table S3. Pairwise F_{ST} values of microsatellites across 10 populations in this study. Significant level α=0.0011 after Bonferroni correction (0.05/45 pairs). The bold indicating significance.

	KS	HN	XZ	SC	YN	SD	CA	ZJ	AH	TJ
KS	0.000									
HN	0.123	0.000								
XZ	**0.283**	0.034	0.000							
SC	**0.323**	0.166	**0.278**	0.000						
YN	**0.320**	0.140	**0.351**	0.103	0.000					
SD	**0.303**	**0.176**	**0.287**	0.105	0.095	0.000				
CA	0.138	0.166	**0.273**	**0.258**	**0.220**	**0.254**	0.000			
ZJ	**0.292**	**0.232**	**0.261**	**0.172**	**0.134**	**0.218**	**0.278**	0.000		
AH	**0.372**	0.127	0.137	**0.168**	**0.176**	**0.196**	**0.299**	**0.248**	0.000	
TJ	**0.361**	**0.184**	0.101	**0.198**	**0.177**	**0.191**	**0.340**	**0.253**	0.093	0.000

Table S4. RAD sequencing quality statistics

Sample ID	Clean Reads	Clean Base	GC (%)	Q30(%)
AH_1	18933617	5594733057	43.63	92.89
AH_2	14260467	4228477829	43.67	92.84
AH_3	20166563	5938749168	43.78	92.97
AH_4	18550176	5463008232	43.88	92.93
AH_5	19123211	5612716006	43.72	92.95
AH_6	21170405	6213330742	43.76	92.94
AH_7	17783993	5218760643	43.75	93.18
AH_8	18109856	5314237612	43.78	92.97
AH_9	20693378	6073429041	43.64	93.15
AH_10	15990704	4691588395	43.77	93.27
XZ_1	21054450	6222738702	43.76	93.46
XZ_2	11091992	3280508002	43.53	93.96
XZ_3	18329298	5397211555	43.81	93.53
XZ_4	24558908	7234682860	43.86	93.51
XZ_5	19282954	5659966963	43.92	93.5
XZ_6	27562649	8091983871	43.69	93.51
XZ_7	23062761	6769830194	43.76	93.68
XZ_8	13725211	4027314467	43.87	93.56
XZ_9	16726801	4908925078	43.8	93.76
XZ_10	15611330	4582169415	43.81	93.78
TJ_1	9282114	2741787728	43.72	93.37
TJ_2	9295428	2747626621	43.61	93.86
TJ_3	12923413	3802045533	43.51	93.81
TJ_4	8993001	2646657488	43.83	93.41
TJ_5	9257081	2715549051	43.72	93.41
TJ_6	8635605	2541723312	44.29	93.12
TJ_7	11715911	3465700376	43.88	93.38
TJ_8	19296466	5670593211	43.69	93.49

Table S4. sequencing quality statistics（continued）

Sample ID	Clean Reads	Clean Base	GC (%)	Q30(%)
TJ_9	18292481	5358215732	43.78	93.47
TJ_10	15562261	4557144277	43.74	93.49
JZ_1	20808325	6108521853	43.73	93.4
JZ_2	17411987	5110119042	43.92	93.41
JZ_3	17553086	5148087099	44.14	93.34
JZ_4	19941391	5852211657	43.65	93.61
JZ_5	17208648	5051755785	43.87	93.62
JZ_6	20656440	6100469330	44.01	93.01
JZ_7	13875362	4114093278	44.25	92.91
JZ_8	30350918	8897720127	44.21	93.61
JZ_9	26819593	7869614126	44.01	93.84
JZ_10	22364797	6563232834	44.03	93.87
ZJ_1	21878197	6445171345	43.91	93.07
ZJ_2	19540310	5737009972	43.84	93.04
ZJ_3	16097388	4724751944	43.89	93.07
ZJ_4	19513768	5728907068	43.77	93.26
ZJ_5	20303941	5961085130	43.76	93.09
ZJ_6	15898627	4665787279	43.85	93.3
ZJ_7	13991497	4106892902	43.91	93.29
ZJ_8	23645070	6988350243	43.94	92.44
ZJ_9	15589363	4623218678	44.01	92.33
SC_1	21128504	6222158275	43.92	92.5
SC_2	18646197	5472783520	43.88	92.48
SC_3	18629390	5468255156	43.78	92.51
SC_4	18348035	5384925435	43.74	92.89
SC_5	19060646	5594288047	43.77	92.55
SC_6	17203955	5049207269	43.78	92.74
SC_7	15011417	4405711067	43.83	92.82
SC_8	19547171	5774216145	43.7	93.65
SC_9	19620909	5757076985	43.88	93.75
SC_10	16143634	4753209398	43.79	93.75

Table S4. sequencing quality statistics（continued）

Sample ID	Clean Reads	Clean Base	GC (%)	Q30(%)
YN_1	11894561	3498911851	44.28	93.66
YN_2	11720290	3436643662	44.27	93.63
YN_3	13113933	3847427753	44.08	93.69
YN_4	9379428	2749530396	44.32	93.83
YN_5	18862207	5566714600	43.55	93.43
YN_6	22367859	6578926204	43.76	93.48
YN_7	27760283	8140333211	43.52	93.57
YN_8	19348527	5670718101	43.52	93.5
YN_9	20426130	5987250295	43.64	93.65
YN_10	17427734	5109453821	43.57	93.72
SD_1	18072383	5339991120	43.8	93.39
SD_2	15042932	4414136332	43.99	93.51
SD_3	13602952	4005688793	44.2	93.42
SD_4	18916227	5569640822	44.11	93.39
SD_5	8847789	2623070035	44.14	93.29
SD_6	15976038	4688546859	44.22	93.39
SD_7	13489051	3957861924	44.52	93.56
SD_8	22646202	6644878253	44.6	93.4
SD_9	25664065	7532188845	44.14	93.61
SD_10	18719770	5493107010	44.15	93.66
HN_1	22780579	6,728,193,256	44	93.47
HN_2	10351052	3,066,220,612	44	93.43
HN_3	24203760	7,124,730,417	44	93.53
HN_4	10184812	2,994,388,489	44	93.62
HN_5	12561098	3,680,804,138	44	93.55
HN_6	16882141	4,949,548,448	44	93.54
HN_7	16015052	4,693,308,655	44	93.71
HN_8	13251306	3,883,138,571	44	93.61
HN_9	16714949	4,899,745,929	44	93.76
HN_10	13858774	4,063,126,772	44	93.82

Table S4. sequencing quality statistics（continued）

Sample ID	Clean Reads	Clean Base	GC (%)	Q30(%)
MK_1	10847858	3,195,741,564	44	93.1
MK_2	17142933	5075121063	44.61	92.86
MK_3	22616853	6656452485	43.98	93.06
MK_4	24897667	7329628297	43.99	93.06
MK_5	14966490	4,384,572,014	44	93.12
MK_6	12298038	3,603,441,647	44	93.43
MK_7	16984005	4,976,391,157	44	93.27
MK_8	13648316	4,002,048,923	44	93.1
MK_9	12977373	3,801,778,235	44	93.35
MK_10	12111238	3545652255	43.74	93.71

Table S5. Coverage depth and degree statistics of RAD-seq of bullfrog samples

Sample ID	Covered Bases(bp)	Coverage 1X（%）	Coverage 5X（%）	Average Depth (x)
AH_1	1,299,132,220	20.78	5.52	0.88
AH_2	1,023,283,503	16.37	4.09	0.67
AH_3	1,357,359,785	21.72	5.96	0.94
AH_4	1,266,829,971	20.27	5.43	0.86
AH_5	1,296,638,676	20.75	5.59	0.89
AH_6	1,391,521,366	22.26	6.22	0.98
AH_7	1,227,933,775	19.65	5.14	0.83
AH_8	1,247,925,390	19.97	5.28	0.84
AH_9	1,366,883,282	21.87	6.09	0.96
AH_10	1,097,812,925	17.56	4.46	0.73
XZ_1	1,237,098,614	19.79	6.63	0.98
XZ_2	859,306,977	13.75	3.11	0.52
XZ_3	1,115,365,182	17.84	5.72	0.85
XZ_4	1,357,763,401	21.72	7.78	1.14
XZ_5	1,154,132,046	18.47	6	0.9
XZ_6	1,457,052,424	23.31	8.73	1.28
XZ_7	1,323,044,572	21.17	7.2	1.07
XZ_8	900,384,378	14.41	4.17	0.64
XZ_9	1,038,871,397	16.62	5.16	0.78
XZ_10	978,951,665	15.66	4.8	0.73
TJ_1	881,981,999	14.11	2.01	0.43
TJ_2	908,948,095	14.54	1.98	0.43
TJ_3	1,220,533,743	19.53	2.86	0.6
TJ_4	865,743,900	13.85	1.88	0.42
TJ_5	878,666,906	14.06	1.97	0.43
TJ_6	691,755,646	11.07	2.25	0.4
TJ_7	1,028,091,322	16.45	2.68	0.55
TJ_8	1,529,697,587	24.47	4.9	0.9
YJ_9	1,459,836,330	23.36	4.58	0.85
TJ_10	1,277,866,878	20.44	3.81	0.72

Table S5. Coverage depth and degree statistics of RAD-seq of bullfrog samples（continued）

Sample ID	Covered Bases(bp)	Coverage 1X（%）	Coverage 5X（%）	Average Depth (x)
JZ_1	1,516,701,403	24.27	5.69	0.97
JZ_2	1,430,667,976	22.89	4.27	0.81
JZ_3	1,417,361,824	22.68	4.45	0.81
JZ_4	1,514,169,877	24.23	5.3	0.93
JZ_5	1,334,991,428	21.36	4.57	0.8
JZ_6	1,346,059,988	21.54	6.17	0.97
JZ_7	953,670,571	15.26	4.14	0.65
JZ_8	1,693,215,793	27.09	9.28	1.41
JZ_9	1,527,762,596	24.44	8.25	1.25
JZ_10	1,356,823,263	21.71	6.86	1.04
ZJ_1	1,404,121,930	22.46	6.63	1.02
ZJ_2	1,278,524,897	20.46	5.83	0.91
ZJ_3	1,115,671,925	17.85	4.64	0.75
ZJ_4	1,281,349,350	20.5	5.79	0.91
ZJ_5	1,317,959,299	21.09	6.09	0.94
ZJ_6	1,107,243,901	17.71	4.58	0.74
ZJ_7	994,179,307	15.91	3.99	0.65
ZJ_8	1,340,853,195	21.45	7.01	1.06
ZJ_9	985,044,316	15.76	4.81	0.73
ZJ_10	1,062,699,747	17	5.23	0.79
SC_1	1,299,990,709	20.8	6.49	0.98
SC_2	1,173,278,073	18.77	5.72	0.87
SC_3	1,170,783,594	18.73	5.68	0.87
SC_4	1,165,701,265	18.65	5.55	0.85
SC_5	1,195,450,345	19.13	5.83	0.89
SC_6	1,103,178,705	17.65	5.2	0.8
SC_7	990,184,965	15.84	4.5	0.7
SC_8	1,277,032,504	20.43	5.85	0.91
SC_9	1,259,562,915	20.15	5.89	0.91
SC_10	1,079,521,852	17.27	4.76	0.75

Table S5. Coverage depth and degree statistics of RAD-seq of bullfrog samples（continued）

Sample ID	Covered Bases(bp)	Coverage 1X（%）	Coverage 5X（%）	Average Depth (x)
YN_1	886,262,824	14.18	3.29	0.55
YN_2	870,505,345	13.93	3.28	0.54
YN_3	936,048,092	14.98	3.73	0.61
YN_4	728,272,156	11.65	2.5	0.44
YN_5	1,508,313,169	24.13	4.79	0.88
YN_6	1,695,222,720	27.12	5.9	1.04
YN_7	1,948,146,166	31.17	7.66	1.29
YN_8	1,525,004,753	24.4	4.92	0.9
YN_9	1,568,834,109	25.1	5.23	0.95
YN_10	1,408,960,608	22.54	4.34	0.81
SD_1	1,392,601,593	22.28	4.85	0.84
SD_2	1,196,764,132	19.15	3.88	0.7
SD_3	1,081,891,204	17.31	3.54	0.63
SD_4	1,418,946,535	22.7	5.17	0.88
SD_5	767,271,934	12.28	2.15	0.41
SD_6	1,236,602,296	19.78	4.26	0.74
SD_7	1,082,065,792	17.31	3.63	0.63
SD_8	1,559,791,479	24.96	6.72	1.05
SD_9	1,744,283,524	27.91	7.48	1.19
SD_10	1,404,674,340	22.47	5.11	0.87
HN_1	1,151,565,362	18.42	7.42	1.06
HN_2	595,783,643	9.53	3.38	0.49
HN_3	1,188,623,258	19.02	7.9	1.13
HN_4	610,802,980	9.77	3.29	0.47
HN_5	718,100,949	11.49	4.07	0.58
HN_6	901,397,795	14.42	5.45	0.78
HN_7	876,774,657	14.03	5.17	0.74
HN_8	755,189,401	12.08	4.28	0.61
HN_9	907,487,470	14.52	5.38	0.78
HN_10	778,292,088	12.45	4.46	0.64

Table S5. Coverage depth and degree statistics of RAD-seq of bullfrog samples（continued）

Sample ID	Covered Bases(bp)	Coverage 1X（%）	Coverage 5X（%）	Average Depth（x）
MK_1	651,646,573	10.43	3.35	0.51
MK_2	882,554,745	14.12	5.4	0.8
MK_3	1,112,808,057	17.8	7.2	1.05
MK_4	1,185,818,570	18.97	7.9	1.16
MK_5	828,317,089	13.25	4.71	0.69
MK_6	882,445,030	14.12	3.39	0.56
MK_7	919,059,465	14.7	5.3	0.79
MK_8	744,954,663	11.92	4.34	0.63
MK_9	748,328,820	11.97	4.09	0.6
MK_10	878,626,426	14.06	3.31	0.56

Table S6. SNP functional information in genome region of bullfrog

Type	Count	Percent
3_prime_UTR_variant	58,714	0.17%
5_prime_UTR_premature_start_codon_gain_variant	6,064	0.02%
5_prime_UTR_variant	35,933	0.10%
downstream_gene_variant	646,262	1.86%
intergenic_region	31,936,431	91.77%
intragenic_variant	199	0.00%
intron_variant	1,341,559	3.86%
missense_variant	67,111	0.19%
non_coding_transcript_exon_variant	23	0.00%
non_coding_transcript_variant	523	0.00%
splice_acceptor_variant	328	0.00%
splice_donor_variant	396	0.00%
splice_region_variant	8,257	0.02%
start_lost	314	0.00%
stop_gained	2,433	0.01%
stop_lost	289	0.00%
stop_retained_variant	112	0.00%
synonymous_variant	47,323	0.14%
upstream_gene_variant	649,594	1.87%

3_prime_UTR_variant: Number and proportion of SNPs in the 3' untranslated region (3'UTR).
5_prime_UTR_premature_start_codon_gain_variant: Number and proportion of SNPs in the 5' untranslated region (5'UTR) that result in the gain of a premature start codon.
5_prime_UTR_variant: Number and proportion of SNPs in the 5' untranslated region (5'UTR).
Downstream_gene_variant: Number and proportion of SNPs in the downstream region of a gene.
Intergenic_region: Number and proportion of SNPs in the region between genes.
Intragenic_variant: Number and proportion of SNPs in non-functional regions within a gene.
Intron_variant: Number and proportion of SNPs in intronic regions (regions within the gene but not part of the coding sequence).
Missense_variant: Number and proportion of SNPs in exonic regions that result in a change in the amino acid sequence (non-synonymous mutation).
Non_coding_transcript_exon_variant: Variant in exonic regions of non-coding transcripts.
Non_coding_transcript_variant: Variant in non-coding transcripts.
Splice_acceptor_variant: Number and proportion of SNPs at the junction region on the left side of an intron.
Splice_donor_variant: Number and proportion of SNPs at the junction region on the right side of an intron.
Splice_region_variant: Number and proportion of SNPs within 2 base pairs of exon-intron junctions.
Start_lost: Number and proportion of SNPs that result in the loss of the start codon.
Stop_gained: Number and proportion of SNPs that result in the gain of a stop codon.
Stop_lost: Number and proportion of SNPs that result in the loss of a stop codon.

Stop_retained_variant: Number and proportion of SNPs that result in a change in the coding sequence of a stop codon.
Synonymous_variant: Number and proportion of SNPs that result in a synonymous mutation (no change in the amino acid sequence).
Upstream_gene_variant: Number and proportion of SNPs in the upstream region of a gene.

Table S7. Genomic regions and candidate genes identified by top 1% Fst for different populations of bullfrogs with RAD-seq

	scaffold	target	start	end	gene
pop1 vs pop2, pop3	KX686108.1	gene	2948	3908	ID=gene22209;
					Name=nad1
	KX686108.1	gene	3909	3979	ID=gene22210;
					Name=trnI
	KX686108.1	gene	3980	4050	ID=gene22211;
					Name=trnQ
	KV980795.1	gene	1473	16730	ID=gene14184;
					Name=AB205_0095820
	KV973396.1	gene	7607	18207	ID=gene14316;
					Name=AB205_0183750
	KZ061044.1	gene	1	1363	ID=gene17650;
					Name=AB205_0001660
	KV924641.1	gene	264534	282451	ID=gene1243;
					Name=AB205_0156310
pop2 vs pop1, pop3	KX686108.1	gene	2948	3908	ID=gene22209; Name=nad1
	KX686108.1	gene	3909	3979	ID=gene22210; Name=trnI
	KX686108.1	gene	3980	4050	ID=gene22211; Name=trnQ
pop3 vs pop1, pop2	KX686108.1	gene	2948	3908	ID=gene22209; Name=nad1
	KX686108.1	gene	3909	3979	ID=gene22210; Name=trnI
	KX686108.1	gene	3980	4050	ID=gene22211; Name=trnQ
